# Synthèse et structure cristalline d’un matériau noir AgMn^II^_3_(Mn^III^_0,26_Al_0,74_)(MoO_4_)_5_

**DOI:** 10.1107/S2056989015003345

**Published:** 2015-02-21

**Authors:** Chahira Bouzidi, Wafa Frigui, Mohamed Faouzi Zid

**Affiliations:** a Laboratoire de Matériaux et Cristallochimie, Faculté des Sciences de Tunis, Université de Tunis ElManar, 2092 Manar II Tunis, Tunisie

**Keywords:** crystal structure, aluminium, manganese, molybdate, solid-state methods, physicochemical properties

## Abstract

A new silver aluminium trimangan­ese penta­molybdate {silver(I) trimanganese(II) aluminium penta­kis­[tetra­oxidomolybdate(VI)]}, AgMn^II^_3_(Mn^III^_0,26_Al_0,74_)(MoO_4_)_5_, has been synthesized using solid-state methods. The structure is composed of *M*_2_O_10_ dimers, *M*_3_O_14_ (*M* = Mn, Al) trimers and MoO_4_ tetra­hedra sharing corners and forming three types of layers *A*, *B* and *B*′. The sequence of the constituting layers is *A*–*BB*′–*A*–*BB*′, with *B*′ obtained from *B* by inversion symmetry, forming a three-dimensional structure with large channels in which the positionally disordered and partially occupied Ag^+^ ions reside. The Mn^III^ and Al^III^ atoms share the same site, *M*. AgMn^II^_3_(Mn^III^_0,26_Al_0,74_)(MoO_4_)_5_ is isotypic with the NaMg_3_ *X*(MoO_4_)_5_ (*X* = Al, In) family and with NaFe_4_(MoO_4_)_5_. A comparative structural description is provided between the structure of the title compound and those of related phases containing dimers, trimers and tetra­mers.

## Abstract 2

A new silver aluminium trimangan­ese penta­molybdate, AgMn^II^_3_(Mn^III^_0,26_Al_0,74_)(MoO_4_)_5_, is composed of *M*_2_O_10_ dimers, *M*_3_O_14_ (*M* = Mn, Al) trimers and MoO_4_ tetra­hedra sharing corners and forming three types of layers. A comparative structural description is provided with the structures of related phases containing dimers, trimers and tetra­mers.

## Contexte chimique   

Les matériaux inorganiques à charpentes ouvertes formées d’octa­èdres et de tétraèdres ont un champ prometteur pour nombreuses applications: conduction ionique (Amine *et al.*, 2000 ▸; Padhi *et al.*, 1997 ▸; Li *et al.*, 2002 ▸) et propriétés magnétiques (Choi & Hong, 2005 ▸).

Dans le but de synthétiser un matériau de formulation analogue à NaMg_3_Al(MoO_4_)_5_ (Hermanowicz *et al.*, 2006 ▸) présentant des propriétés physiques intéressantes, nous avons exploré le système Ag_2_O—MnO—Al_2_O_3_—MoO_3_. C’est dans ce cadre, que nous avons pu synthétisé une nouvelle phase, par réaction à l’état solide, de formulation AgMn^II^_3_(Mn^III^_0,26_Al_0,74_)(MoO_4_)_5_.

## Commentaire structurelle   

Dans la charpente anionique, les octa­èdres *M*1O_6_, *M*2O_6_ et *M*4O_6_ (*M* = Mn, Al) partagent deux arêtes pour former un trimère de type *M*_3_O_14_. Par contre l’octa­èdre *M*3O_6_ se lie avec son homologue par mise en commun d’une arête pour conduire à un dimère *M*3_2_O_10_. La jonction des dimères et des trimères, réalisée par mise en commun de sommets avec les tétraèdres MoO_4_, conduit à une structure tridimensionnelle.

**Figure 1:**
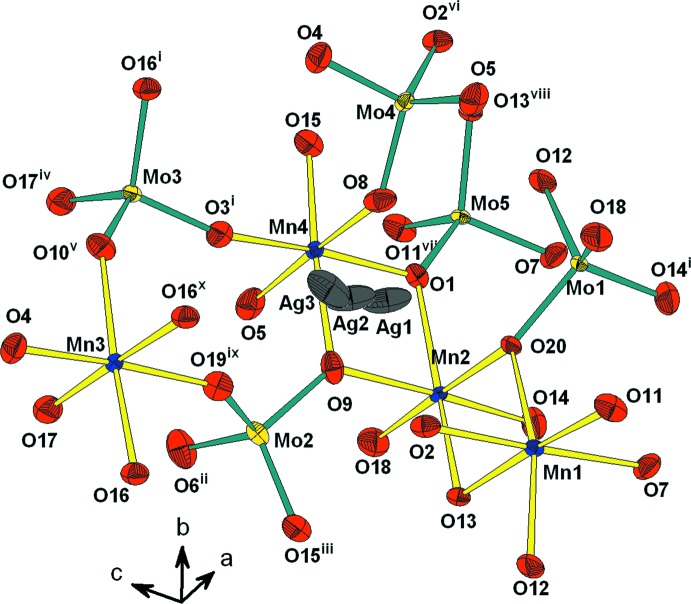
Représentation de l’unité asymétrique dans AgMn^II^_3_(Mn^III^_0,26_Al_0,74_)(MoO_4_)_5_. Les éllipsoïdes ont été définis avec 50% de probabilité. [Code de symétrie: (i) −*x* + 1, −*y* + 1, −*z* + 1; (ii) *x* + 1, *y*, *z*; (iii) *x*, *y* + 1, *z*; (iv) *x* − 1, *y*, *z*; (v) −*x* + 1, −*y* + 1, −*z* + 2; (vi) −*x* + 1, −*y* + 2, −*z* + 1; (vii) *x* + 1, *y* − 1, *z*; (viii) *x*, *y* − 1, *z*; (ix) *x*, *y*, *z* + 1; (x) *x*, *y* + 1, *z* + 1.]

**Figure 2:**
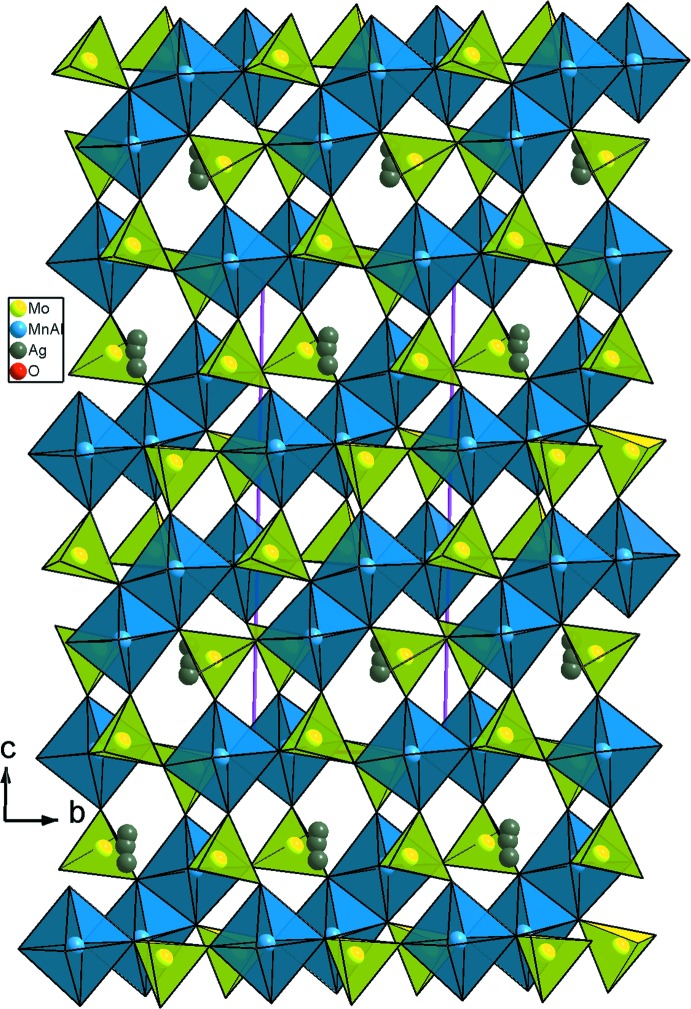
Projection de la structure de AgMn^II^_3_(Mn^III^_0,26_Al_0,74_)(MoO_4_)_5_, selon *c*.

**Figure 3:**
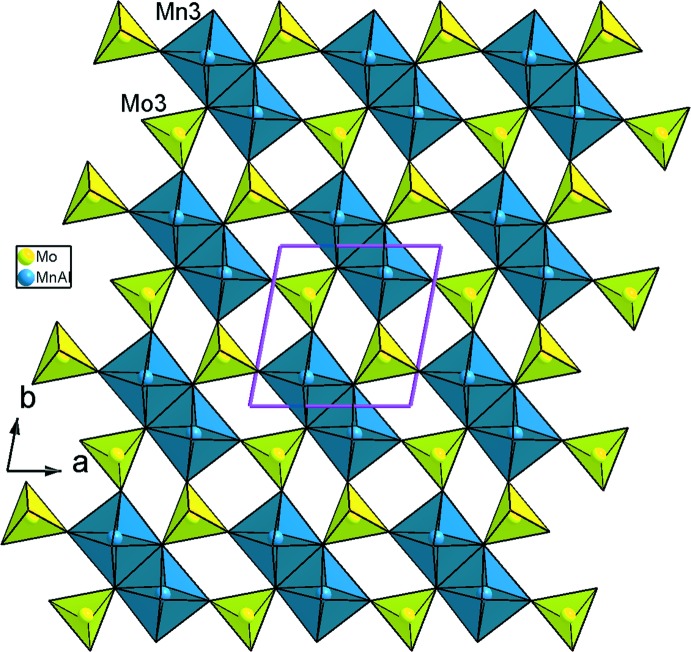
Représentation d’une couche de type *A*, selon [001], dans AgMn^II^_3_(Mn^III^_0,26_Al_0,74_)(MoO_4_)_5_.

**Figure 4:**
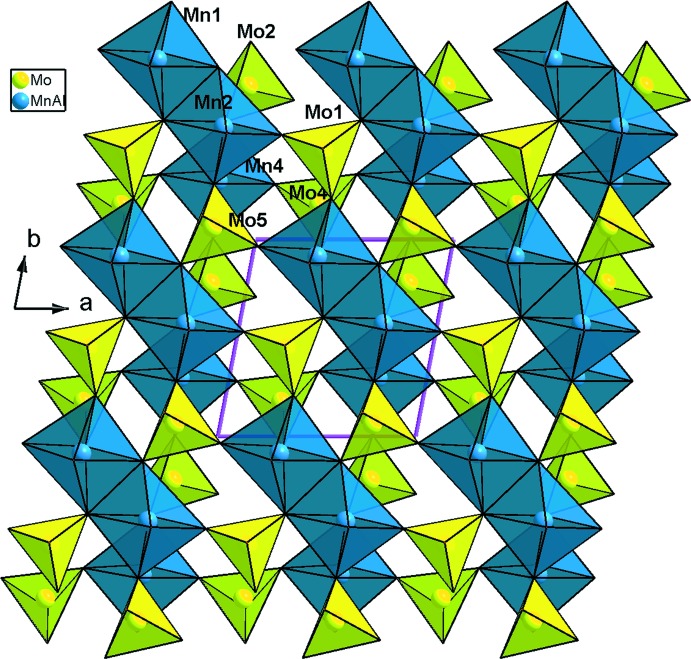
Représentation de couches de type *B*, selon [001], dans AgMn^II^_3_(Mn^III^_0,26_Al_0,74_)(MoO_4_)_5_.

**Figure 5:**
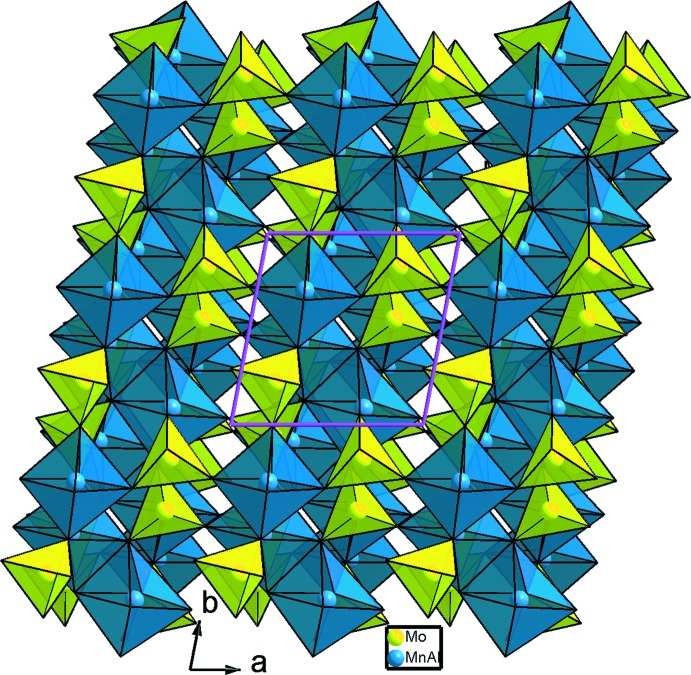
Représentation des doubles couches *BB*′, montrant leur jonction selon *a*.

**Figure 6:**
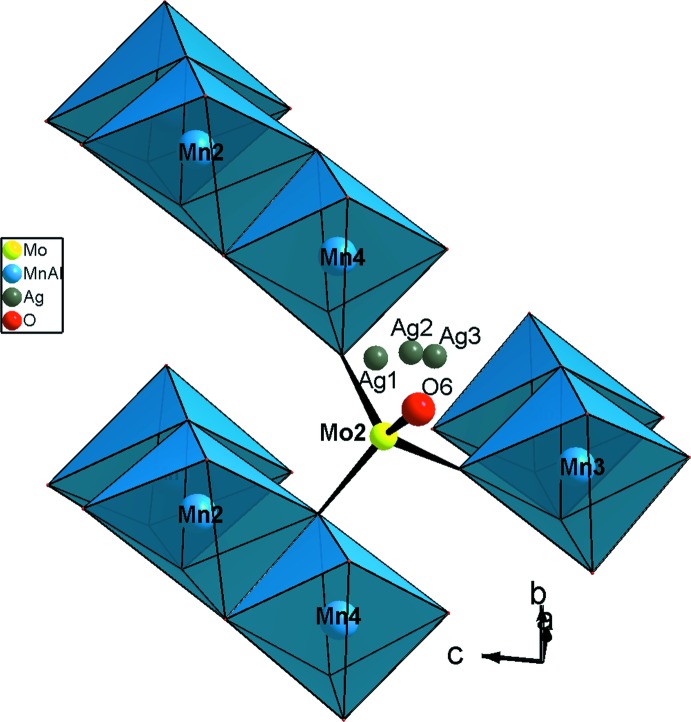
Environnement du tétraèdre Mo_2_O_4_ mettant en évidence le groupement molybdyl (Mo—O_L_).

L’unité asymétrique est construite par un trimère *M*_3_O_14_ (*M* = Mn/Al), un octa­èdre *M*3O_6_ (*M*3 = Mn3/Al3) et cinq tétraèdres MoO_4_ liés par mise en commun des sommets. La compensation de charge est assurée par les ions Ag^+^ repartit statistiquement sur trois sites proches (Fig. 1 ▸). Un examen rigoureux de la charpente anionique tridimensionnelle révèle qu’elle est construite d’un assemblage de couches disposées parallèlement au plan (001) et sont reliées par formation des ponts mixtes Mo—O—*M* (Fig. 2 ▸). En effet, on distingue deux types de couches *A* et *B*. Les couches de type *A* sont formées par les dimères *M*3_2_O_10_ et les tétraèdres Mo3O_4_ liés par partage de sommets (Fig. 3 ▸). Par contre, les couches de type *B* sont construites par les trimères *M*_3_O_14_ et les tétraèdres Mo1O_4_, Mo2O_4_, Mo4O_4_ et Mo5O_4_ connectés par partage de sommets (Fig. 4 ▸). Ces couches (type *B*) sont connectées à des autres centrosymétriques et adjacentes notées *B*′, par formation des ponts mixtes de types Mo1—O—*M* et Mo5—O—*M* (*M* = Mn, Al), pour donner des bicouches *BB*′ (Fig. 5 ▸). Il en résulte une disposition alternée des différentes couches de type *A*—*BB*′—*A*—*BB*′, regroupées par formation de ponts de type Mo2—O—*M*, Mo4—O—*M* et Mo3—O—*M* ce qui conduit à une structure tridimensionnelle possédant des canaux où logent les atomes d’argent mais excentrés (Fig. 2 ▸). Il est à signaler que le quatrième sommet dans chaque tétraèdre Mo2O_4_, restant libre, forme un groupement molybdyl (Mo—O_L_) et pointe vers le canal où résident les cations Ag^+^ (Fig. 6 ▸).

Dans chacun des tétraèdres, on relève des distances moyennes, *d*(Mo—O) de l’ordre de 1,768 (2) Å (Tableau 1 ▸), semblables à celles observées dans la bibliographie (Solodovnikov *et al.*, 1997 ▸; Sarapulova *et al.*, 2009 ▸; Ennajeh *et al.*, 2013 ▸). D’autre part, les distances moyennes, *d*(*M*—O) dans les octa­èdres *M*O_6_ (*M* = Mn, Al) s’avèrent une moyenne entre celles *d*(Mn^II^—O) et *d*(Al—O) rencontrées dans la littérature (Moring & Kostiner, 1986 ▸; Hatert, 2006 ▸). En effet, on remarque aussi qu’elles varient en fonction du taux d’occupation de l’aluminium dans les sites.

Les distances inter­atomiques Ag—O varient de 2,242 (4) à 2,539 (6) Å, ce qui est conforme à celles observées dans de nombreux composés retrouvés dans la bibliographie (Kacimi *et al.*, 2005 ▸; Balsanova *et al.*, 2009 ▸).

De plus, le calcul des valences de liaison (BVS), utilisant la formule empirique de Brown (Brown & Altermatt, 1985 ▸), conduit aux valeurs des charges des cations suivants: Ag1 (1,13), Ag2 (0,91), Ag3 (0,79), Mo1 (5,88), Mo2 (6,00), Mo3 (6,01), Mo4 (6,09), Mo5 (6,00) et en incluant les taux d’occupation des sites *M*, on trouve que la somme des différentes valeurs calculées (+9,04): Mn1/Al1 (2,004), Mn2/Al2 (2,38), Mn3/Al3 (2,05), Mn4/Al4 (2,60), confirme bien la charge globale (+9) apportée par les ions Mn et Al dans la formule AgMn^II^_3_(Mn^III^_0,26_Al_0,74_)(MoO_4_)_5_.

## Enquête de base de données   

**Figure 7:**
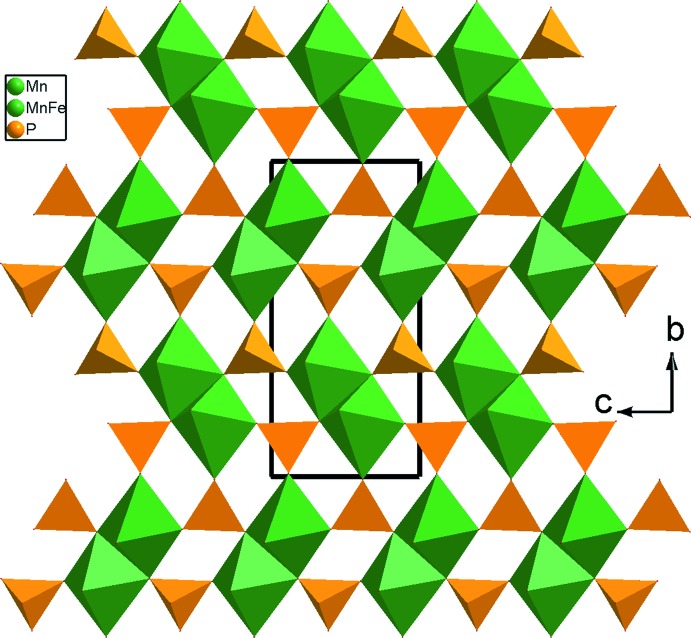
Représentation d’une couche, selon *a*, dans Na_2_FeMn_2_(PO_4_)_3_ montrant la disposition des dimères *M*_2_O_12_ (*M* = Mn, Fe).

**Figure 8:**
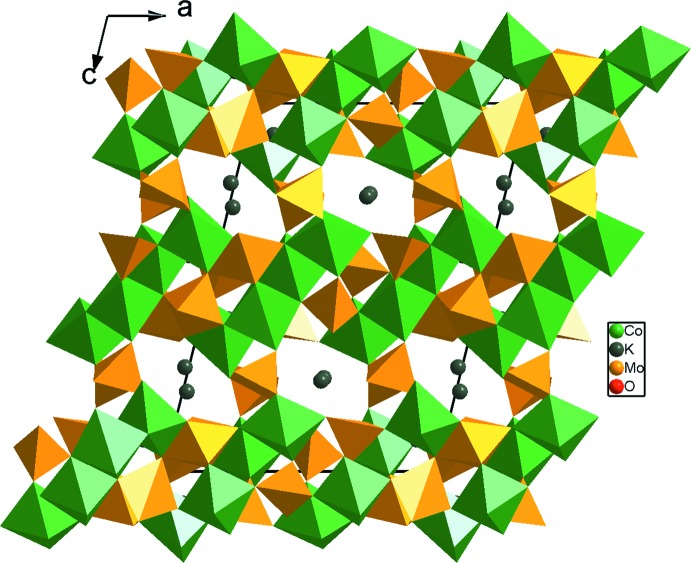
Projection de la structure de K_2_Co_2_Mo_3_O_12_, selon *b*, mettant en évidence les tétramères Co_4_O_18_.

Un examen bibliographie montre que la phase synthétisée est isostructurale à celles de formulation NaFe_4_(MoO_4_)_5_ (Muessig *et al.*, 2003 ▸), NaMg_3_Al(MoO_4_)_5_ (Hermanowicz *et al.*, 2006 ▸) et NaMg_3_In(MoO_4_)_5_ (Klevtsova *et al.*, 1993 ▸). La recherche de structures présentant des aspects communs avec celle de AgMn^II^_3_(Mn^III^_0,26_Al_0,74_)(MoO_4_)_5_, nous a conduit à la famille des alluaudites et plus précisément le composé Na_2_FeMn_2_(PO_4_)_3_ (Daidouh *et al.*, 2002 ▸) possédant des dimères dans les couches. Une différence nette dans la disposition des dimères a été observée. En effet, dans Na_2_FeMn_2_(PO_4_)_3_ les dimères *M*_2_O_12_ (*M* = Mn, Fe) sont disposés d’une façon perpendiculaire (Fig. 7 ▸), contrairement à notre structure où ils sont parallèles les uns aux autres (Fig. 3 ▸). La comparaison de notre structure avec le matériau K_2_Co_2_Mo_3_O_12_ (Engel *et al.*, 2009 ▸) montre une différence nette dans l’arrangement des octa­èdres. En effet, dans K_2_Co_2_Mo_3_O_12_ les octa­èdres CoO_6_ partagent trois arêtes pour former les tétramères Co_4_O_18_. Ces derniers sont inter­connectés les uns aux autres moyennant les tétraèdres MoO_4_ par mise en commun des sommets afin de conduire à une structure tridimensionnelle possédant des canaux où résident les cations K^+^ (Fig. 8 ▸).

**Figure 9:**
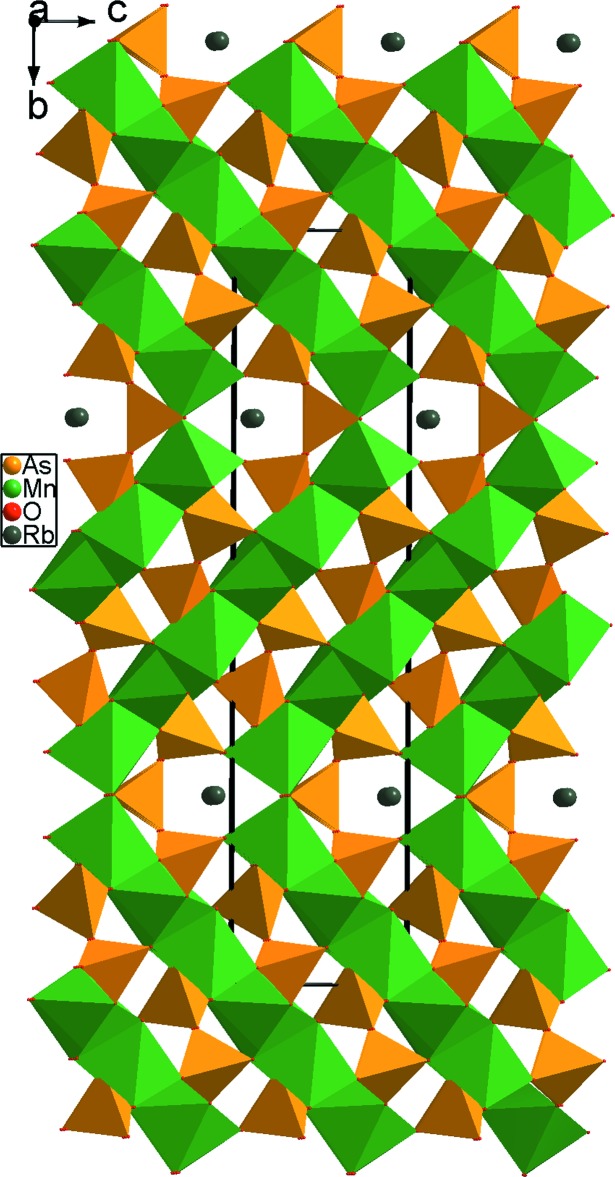
Projection de la structure de RbMn_6_(As_2_O_7_)_2_(As_3_O_10_), montrant les chaînes d’octa­èdres MnO_6_ disposées en zigzag.

De plus, la comparaison de la structure étudiée avec celle du composé RbMn_6_(As_2_O_7_)_2_(As_3_O_10_) (Ayed *et al.*, 2004 ▸) montre que dans cette dernière les octa­èdres MnO_6_ se connectent entre eux, toujours, par mise en commun d’arêtes pour former des chaînes d’octa­èdres disposées en zigzag. Ces dernières sont liées aux tétraèdres AsO_4_ pour donner une structure tridimensionnelle (Fig. 9 ▸).

## Synthèse et cristallisation   

**Figure 10:**
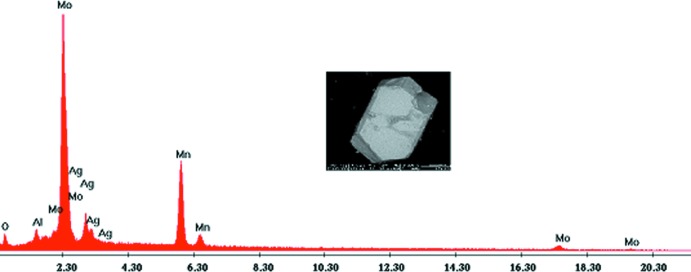
Spectre d’analyse qualitative et morphologie d’un cristal de AgMn^II^_3_(Mn^III^_0,26_Al_0,74_)(MoO_4_)_5_.

Afin de trouver une nouvelle phase de formulation analogue à NaMg_3_Al(MoO_4_)_5_, nous avons pu synthétiser le matériau AgMn^II^_3_(Mn^III^_0,26_Al_0,74_)(MoO_4_)_5_. Les réactifs, Al_2_O_3_ (Fluka, 06285), AgNO_3_ (Fluka, 85230), C_9_H_9_MnO_6_·2H_2_O (Fluka, 63538) et (NH_4_)_2_Mo_4_O_13_ (Fluka, 69858) sont pris dans les proportions Al:Ag:Mn:Mo égales à 1:1:3:5 dans un creuset en porcelaine. Le mélange finement broyé, est préchauffe dans un four jusqu’à 623 K en vue d’éliminer les composés volatils. Il est ensuite porté jusqu’à une température de synthèse proche de celle de la fusion à 1143 K. Le produit est alors abandonné à cette température pendant 4 semaines pour favoriser la germination et la croissance des cristaux. Le résidu final a subi en premier lieu un refroidissement lent (5°/12 h) jusqu’à 1043 K puis rapide (50°/h) jusqu’à la température ambiante. Des cristaux de couleur noir, ont été séparés du flux par l’eau chaude. Une analyse qualitative au MEB de marque FEI et de type Quanta 200 confirme la présence des différents éléments chimiques attendus: Mo, Mn, Ag, Al et l’oxygène (Fig. 10 ▸).

## Affinement   

Détails de donnés crystallines, collection de donnés et affinement sont résumés dans le Tableau 2 ▸. La structure a été résolu par la méthode directe *SHELXS97* (Sheldrick, 2008 ▸), partant de la formule AgAlMn_3_Mo_5_O_20_ similaire au composé isotype NaAlMg_3_Mo_5_O_20_. Un examen de la Fourier différence montre des anomalies autour des ions Mn^2+^ et Ag^+^. L’affinement, et en se basant sur les facteurs géométrique, a été mené d’une part avec les taux d’occupation variables pour Mn et Al occupant statiquement les mêmes positions et ayant les mêmes ellipsoïdes utilisant les deux fonctions EXYZ et EADP autorisées par *SHELXL97* (Sheldrick, 2008 ▸), et d’autre part en considérant que l’ion Ag^+^ est reparti sur trois positions proches dans la structure. En effet, l’affinement de tous les paramètres variables conduit à des ellipsoïdes bien définis. Les densités d’électrons maximum et minimum restants dans la Fourier-différence sont situées respectivement à 0,73 Å de Mo1 et à 0,85 Å de Mo1. Il en résulte, la composition chimique finale, Ag_0,986_Mn^II^_3_(Mn^III^_0,261_Al_0,739_)(MoO_4_)_5_ du nouveau matériau obtenu.

## Supplementary Material

Crystal structure: contains datablock(s) I. DOI: 10.1107/S2056989015003345/ru2061sup1.cif

Structure factors: contains datablock(s) I. DOI: 10.1107/S2056989015003345/ru2061Isup2.hkl

CCDC references: 1049452, 1050266

Additional supporting information:  crystallographic information; 3D view; checkCIF report

## Figures and Tables

**Table 1:** Longueurs des liaisons slectionnes ()

Mo1O14^i^	1.729(4)	Mn2O14	2.084(4)
Mo1O18	1.737(4)	Mn2O13	2.096(3)
Mo1O12	1.747(4)	Mn2O1	2.121(3)
Mo1O20	1.796(3)	Mn2O20	2.148(3)
Mo2O6^ii^	1.717(4)	Mn2O9	2.164(4)
Mo2O19	1.749(3)	Mn3O4	2.037(4)
Mo2O15^iii^	1.758(4)	Mn3O17	2.043(4)
Mo2O9	1.819(3)	Mn3O19^ix^	2.079(4)
Mo3O17^iv^	1.737(4)	Mn3O10	2.105(4)
Mo3O10^v^	1.748(3)	Mn3O16^i^	2.126(4)
Mo3O3^i^	1.751(4)	Mn3O16^x^	2.160(4)
Mo3O16^i^	1.795(3)	Mn4O3	1.985(4)
Mo4O4	1.732(4)	Mn4O5^ii^	2.016(4)
Mo4O5^i^	1.744(4)	Mn4O1	2.035(3)
Mo4O2^vi^	1.765(3)	Mn4O15	2.036(4)
Mo4O8^i^	1.792(4)	Mn4O8	2.068(4)
Mo5O11^vii^	1.721(3)	Mn4O9	2.131(4)
Mo5O7	1.721(3)	Ag1O8	2.242(4)
Mo5O13^viii^	1.781(3)	Ag1O2	2.260(4)
Mo5O1	1.808(3)	Ag1O6	2.275(4)
Mn1O12^iii^	2.094(4)	Ag2O6	2.255(9)
Mn1O11	2.114(3)	Ag2O8	2.388(13)
Mn1O20	2.150(3)	Ag2O2	2.514(12)
Mn1O7^i^	2.158(4)	Ag3O6	2.245(5)
Mn1O2	2.161(3)	Ag3O8	2.519(5)
Mn1O13	2.188(3)	Ag3O19	2.539(5)
Mn2O18^ii^	2.059(4)		

Symmetry codes: (i) 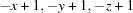; (ii) 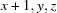; (iii) 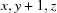; (iv) 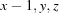; (v) 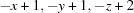; (vi) 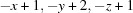; (vii) 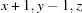; (viii) 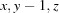; (ix) 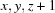; (x) 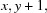; (ix) 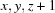; (x) 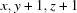.

**Table 2:** Dtails exprimentaux

Donnes crystallines	
Formule chimique	AgAl_0.74_Mn_3.26_Mo_5_O_20_
*M*_r_	1106.64
Systme cristallin, groupe d’espace	Triclinique, *P* 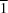
Temprature (K)	298
*a*, *b*, *c* ()	6.9596(6), 7.0326(7), 17.909(6)
, , ()	87.654(6), 87.442(6), 79.299(7)
*V* (^3^)	860.0(3)
*Z*	2
Type de rayonnement	Mo *K*
(mm^1^)	7.08
Taille des cristaux (mm)	0.28 0.21 0.21

Collection de donnes	
Diffractomtre	EnrafNonius CAD-4
Correction d’absorption	scan (North *et al.*, 1968 ▸)
*T*_min_, *T*_max_	0.153, 0.263
Nombre de rflexions mesures, indpendantes et observes [*I* > 2(*I*)]	5479, 3736, 3470
*R*_int_	0.015

Affinement	
*R*[*F* ^2^ > 2(*F* ^2^)], *wR*(*F* ^2^), *S*	0.025, 0.064, 1.23
Nombre de rflexions	3736
Nombre de paramtres	291
Nombre de restraints	1
_max_, _min_ (e ^3^)	1.00, 1.06

Programmes informatiques: *CAD-4 EXPRESS* (Duisenberg, 1992 ▸; Macek Yordanov, 1992 ▸), *XCAD4* (Harms Wocadlo, 1995 ▸), *SHELXS97* et *SHELXL97* (Sheldrick, 2008 ▸), *DIAMOND* (Brandenburg Putz, 2001 ▸) et *WinGX* (Farrugia, 2012 ▸).

## supplementary crystallographic information

### Crystal data

**Table:**

AgAl_0.74_Mn_3.26_Mo_5_O_20_	*Z* = 2
*M**_r_* = 1106.64	*F*(000) = 1016
Triclinic, *P*1	*D*_x_ = 4.274 Mg m^−^^3^
Hall symbol: -P 1	Mo *K*α radiation, λ = 0.71073 Å
*a* = 6.9596 (6) Å	Cell parameters from 25 reflections
*b* = 7.0326 (7) Å	θ = 10–15°
*c* = 17.909 (6) Å	µ = 7.08 mm^−^^1^
α = 87.654 (6)°	*T* = 298 K
β = 87.442 (6)°	Prism, black
γ = 79.299 (7)°	0.28 × 0.21 × 0.21 mm
*V* = 860.0 (3) Å^3^	

### Data collection

**Table:**

Enraf–Nonius CAD-4 diffractometer	3470 reflections with *I* > 2/s(*I*)
Radiation source: fine-focus sealed tube	*R*_int_ = 0.015
Graphite monochromator	θ_max_ = 27.0°, θ_min_ = 2.3°
ω/2θ scans	*h* = −8→3
Absorption correction: ψ scan (North *et al.*, 1968)	*k* = −8→8
*T*_min_ = 0.153, *T*_max_ = 0.263	*l* = −22→22
5479 measured reflections	2 standard reflections every 120 min
3736 independent reflections	intensity decay: 1.1%

### Refinement

**Table:**

Refinement on *F*^2^	Primary atom site location: structure-invariant direct methods
Least-squares matrix: full	Secondary atom site location: difference Fourier map
*R*[*F*^2^ > 2σ(*F*^2^)] = 0.025	*w* = 1/[σ^2^(*F*_o_^2^) + (0.0222*P*)^2^ + 3.8945*P*] where *P* = (*F*_o_^2^ + 2*F*_c_^2^)/3
*wR*(*F*^2^) = 0.064	(Δ/σ)_max_ = 0.001
*S* = 1.23	Δρ_max_ = 1.00 e Å^−^^3^
3736 reflections	Δρ_min_ = −1.06 e Å^−^^3^
291 parameters	Extinction correction: *SHELXL97* (Sheldrick, 2008), Fc^*^=kFc[1+0.001xFc^2^λ^3^/sin(2θ)]^-1/4^
1 restraint	Extinction coefficient: 0.00248 (15)

### Special details

**Table:**

Geometry. All e.s.d.'s (except the e.s.d. in the dihedral angle between two l.s. planes) are estimated using the full covariance matrix. The cell e.s.d.'s are taken into account individually in the estimation of e.s.d.'s in distances, angles and torsion angles; correlations between e.s.d.'s in cell parameters are only used when they are defined by crystal symmetry. An approximate (isotropic) treatment of cell e.s.d.'s is used for estimating e.s.d.'s involving l.s. planes.
Refinement. Refinement of *F*^2^ against ALL reflections. The weighted *R*-factor *wR* and goodness of fit *S* are based on *F*^2^, conventional *R*-factors *R* are based on *F*, with *F* set to zero for negative *F*^2^. The threshold expression of *F*^2^ > σ(*F*^2^) is used only for calculating *R*-factors(gt) *etc*. and is not relevant to the choice of reflections for refinement. *R*-factors based on *F*^2^ are statistically about twice as large as those based on *F*, and *R*- factors based on ALL data will be even larger.

### Fractional atomic coordinates and isotropic or equivalent isotropic displacement parameters (Å^2^)

**Table:**

	*x*	*y*	*z*	*U*_iso_*/*U*_eq_	Occ. (<1)
Mo1	0.25717 (6)	0.44969 (5)	0.41400 (2)	0.01218 (10)	
Mo2	0.81551 (6)	0.77839 (6)	0.18950 (2)	0.01421 (10)	
Mo3	0.22876 (6)	0.69185 (5)	0.97314 (2)	0.01166 (10)	
Mo4	0.71529 (6)	0.82667 (5)	0.78655 (2)	0.01120 (10)	
Mo5	0.77290 (5)	0.06208 (5)	0.403892 (19)	0.00848 (9)	
Mn1	0.32746 (10)	0.91577 (9)	0.38513 (4)	0.0093 (2)	0.957 (7)
Al1	0.32746 (10)	0.91577 (9)	0.38513 (4)	0.0093 (2)	0.043 (7)
Mn2	0.72939 (10)	0.57389 (10)	0.37410 (4)	0.0081 (2)	0.846 (7)
Al2	0.72939 (10)	0.57389 (10)	0.37410 (4)	0.0081 (2)	0.154 (7)
Mn3	0.68693 (10)	0.82604 (10)	0.99326 (4)	0.0088 (2)	0.804 (7)
Al3	0.68693 (10)	0.82604 (10)	0.99326 (4)	0.0088 (2)	0.196 (7)
Mn4	0.75043 (12)	0.30015 (11)	0.23276 (4)	0.0092 (3)	0.654 (7)
Al4	0.75043 (12)	0.30015 (11)	0.23276 (4)	0.0092 (3)	0.346 (7)
Ag1	0.3550 (4)	0.6550 (3)	0.2181 (4)	0.0373 (9)	0.448 (9)
Ag2	0.3820 (17)	0.6607 (11)	0.1843 (13)	0.0373 (9)	0.219 (12)
Ag3	0.3870 (5)	0.6554 (7)	0.1610 (5)	0.0401 (12)	0.319 (12)
O1	0.7352 (5)	0.2824 (5)	0.34651 (18)	0.0153 (6)	
O2	0.3399 (5)	0.9515 (5)	0.26468 (19)	0.0190 (7)	
O3	0.7466 (6)	0.3498 (6)	0.1229 (2)	0.0279 (8)	
O4	0.6928 (6)	0.8788 (5)	0.8806 (2)	0.0249 (8)	
O5	0.0428 (6)	0.2814 (5)	0.2342 (2)	0.0266 (8)	
O6	0.0586 (5)	0.7080 (6)	0.1646 (2)	0.0311 (9)	
O7	0.7079 (6)	0.1205 (5)	0.49512 (19)	0.0228 (8)	
O8	0.4491 (6)	0.3332 (5)	0.23101 (19)	0.0241 (8)	
O9	0.7330 (5)	0.5994 (5)	0.25324 (19)	0.0214 (7)	
O10	0.7026 (5)	0.5284 (5)	0.9776 (2)	0.0231 (8)	
O11	0.0187 (5)	0.9658 (5)	0.3959 (2)	0.0240 (8)	
O12	0.3428 (6)	0.2095 (5)	0.3903 (2)	0.0316 (9)	
O13	0.6472 (5)	0.8752 (5)	0.38069 (17)	0.0137 (6)	
O14	0.7538 (6)	0.5391 (6)	0.4896 (2)	0.0287 (9)	
O15	0.7832 (6)	0.0068 (6)	0.2296 (2)	0.0299 (9)	
O16	0.6236 (5)	0.1365 (5)	0.00392 (19)	0.0195 (7)	
O17	0.9851 (6)	0.7876 (5)	0.9945 (2)	0.0263 (8)	
O18	0.0299 (6)	0.5324 (6)	0.3763 (2)	0.0293 (9)	
O19	0.6781 (5)	0.7961 (5)	0.1093 (2)	0.0224 (7)	
O20	0.4161 (5)	0.6075 (4)	0.37703 (18)	0.0150 (6)	

### Atomic displacement parameters (Å^2^)

**Table:**

	*U*^11^	*U*^22^	*U*^33^	*U*^12^	*U*^13^	*U*^23^
Mo1	0.0156 (2)	0.00828 (18)	0.01359 (19)	−0.00554 (14)	0.00493 (14)	−0.00176 (13)
Mo2	0.01091 (19)	0.0149 (2)	0.0168 (2)	−0.00313 (14)	−0.00111 (14)	0.00465 (14)
Mo3	0.01408 (19)	0.01035 (18)	0.01164 (18)	−0.00514 (14)	−0.00041 (14)	−0.00018 (13)
Mo4	0.0158 (2)	0.00772 (18)	0.00939 (18)	−0.00068 (14)	−0.00021 (14)	0.00003 (13)
Mo5	0.00941 (18)	0.00634 (17)	0.01008 (17)	−0.00234 (13)	−0.00227 (13)	0.00120 (12)
Mn1	0.0082 (4)	0.0068 (3)	0.0124 (4)	−0.0005 (2)	−0.0006 (2)	0.0000 (2)
Al1	0.0082 (4)	0.0068 (3)	0.0124 (4)	−0.0005 (2)	−0.0006 (2)	0.0000 (2)
Mn2	0.0091 (4)	0.0064 (4)	0.0086 (4)	−0.0006 (2)	−0.0008 (2)	−0.0017 (2)
Al2	0.0091 (4)	0.0064 (4)	0.0086 (4)	−0.0006 (2)	−0.0008 (2)	−0.0017 (2)
Mn3	0.0090 (4)	0.0088 (4)	0.0081 (4)	−0.0002 (3)	−0.0013 (3)	−0.0004 (2)
Al3	0.0090 (4)	0.0088 (4)	0.0081 (4)	−0.0002 (3)	−0.0013 (3)	−0.0004 (2)
Mn4	0.0113 (4)	0.0088 (4)	0.0075 (4)	−0.0018 (3)	−0.0011 (3)	−0.0001 (3)
Al4	0.0113 (4)	0.0088 (4)	0.0075 (4)	−0.0018 (3)	−0.0011 (3)	−0.0001 (3)
Ag1	0.0319 (7)	0.0167 (4)	0.063 (3)	0.0045 (4)	−0.0262 (11)	−0.0094 (9)
Ag2	0.0319 (7)	0.0167 (4)	0.063 (3)	0.0045 (4)	−0.0262 (11)	−0.0094 (9)
Ag3	0.0132 (9)	0.0420 (12)	0.066 (3)	−0.0084 (8)	−0.0072 (12)	0.0112 (15)
O1	0.0124 (16)	0.0129 (15)	0.0192 (16)	0.0002 (12)	0.0003 (12)	0.0038 (12)
O2	0.0251 (19)	0.0132 (16)	0.0193 (17)	−0.0043 (14)	−0.0077 (14)	0.0028 (13)
O3	0.040 (2)	0.028 (2)	0.0196 (18)	−0.0153 (17)	0.0037 (16)	−0.0047 (15)
O4	0.031 (2)	0.0255 (19)	0.0175 (17)	−0.0027 (16)	0.0008 (15)	−0.0025 (14)
O5	0.029 (2)	0.0179 (18)	0.028 (2)	0.0061 (15)	0.0028 (16)	−0.0030 (14)
O6	0.0146 (18)	0.046 (2)	0.032 (2)	−0.0043 (17)	−0.0017 (15)	0.0070 (18)
O7	0.032 (2)	0.0220 (18)	0.0149 (16)	−0.0058 (15)	−0.0025 (14)	−0.0046 (13)
O8	0.038 (2)	0.0165 (17)	0.0195 (17)	−0.0078 (16)	−0.0091 (16)	0.0015 (13)
O9	0.0174 (17)	0.0231 (18)	0.0203 (17)	0.0022 (14)	0.0034 (14)	0.0061 (14)
O10	0.0252 (19)	0.0178 (17)	0.0265 (19)	−0.0058 (15)	−0.0009 (15)	0.0062 (14)
O11	0.0108 (16)	0.0208 (18)	0.040 (2)	−0.0014 (14)	−0.0033 (15)	0.0025 (15)
O12	0.040 (2)	0.0141 (18)	0.041 (2)	−0.0119 (17)	0.0188 (19)	−0.0061 (16)
O13	0.0128 (15)	0.0137 (15)	0.0161 (15)	−0.0055 (12)	−0.0028 (12)	−0.0011 (12)
O14	0.035 (2)	0.030 (2)	0.0214 (19)	−0.0086 (17)	0.0069 (16)	0.0020 (15)
O15	0.036 (2)	0.031 (2)	0.027 (2)	−0.0183 (18)	0.0042 (17)	−0.0018 (16)
O16	0.0214 (18)	0.0188 (17)	0.0201 (17)	−0.0073 (14)	−0.0041 (14)	−0.0011 (13)
O17	0.0239 (19)	0.0243 (19)	0.031 (2)	−0.0053 (15)	0.0024 (16)	−0.0056 (15)
O18	0.025 (2)	0.035 (2)	0.030 (2)	−0.0130 (17)	−0.0031 (16)	−0.0034 (17)
O19	0.0211 (18)	0.0210 (18)	0.0237 (18)	−0.0004 (14)	−0.0059 (14)	0.0037 (14)
O20	0.0166 (16)	0.0100 (15)	0.0190 (16)	−0.0052 (12)	0.0052 (13)	−0.0014 (12)

### Geometric parameters (Å, º)

**Table:**

Mo1—O14^i^	1.729 (4)	Mn2—O14	2.084 (4)
Mo1—O18	1.737 (4)	Mn2—O13	2.096 (3)
Mo1—O12	1.747 (4)	Mn2—O1	2.121 (3)
Mo1—O20	1.796 (3)	Mn2—O20	2.148 (3)
Mo2—O6^ii^	1.717 (4)	Mn2—O9	2.164 (4)
Mo2—O19	1.749 (3)	Mn3—O4	2.037 (4)
Mo2—O15^iii^	1.758 (4)	Mn3—O17	2.043 (4)
Mo2—O9	1.819 (3)	Mn3—O19^ix^	2.079 (4)
Mo3—O17^iv^	1.737 (4)	Mn3—O10	2.105 (4)
Mo3—O10^v^	1.748 (3)	Mn3—O16^i^	2.126 (4)
Mo3—O3^i^	1.751 (4)	Mn3—O16^x^	2.160 (4)
Mo3—O16^i^	1.795 (3)	Mn4—O3	1.985 (4)
Mo4—O4	1.732 (4)	Mn4—O5^ii^	2.016 (4)
Mo4—O5^i^	1.744 (4)	Mn4—O1	2.035 (3)
Mo4—O2^vi^	1.765 (3)	Mn4—O15	2.036 (4)
Mo4—O8^i^	1.792 (4)	Mn4—O8	2.068 (4)
Mo5—O11^vii^	1.721 (3)	Mn4—O9	2.131 (4)
Mo5—O7	1.721 (3)	Ag1—O8	2.242 (4)
Mo5—O13^viii^	1.781 (3)	Ag1—O2	2.260 (4)
Mo5—O1	1.808 (3)	Ag1—O6	2.275 (4)
Mn1—O12^iii^	2.094 (4)	Ag2—O6	2.255 (9)
Mn1—O11	2.114 (3)	Ag2—O8	2.388 (13)
Mn1—O20	2.150 (3)	Ag2—O2	2.514 (12)
Mn1—O7^i^	2.158 (4)	Ag3—O6	2.245 (5)
Mn1—O2	2.161 (3)	Ag3—O8	2.519 (5)
Mn1—O13	2.188 (3)	Ag3—O19	2.539 (5)
Mn2—O18^ii^	2.059 (4)		
			
O14^i^—Mo1—O18	111.70 (19)	O18^ii^—Mn2—O14	82.33 (16)
O14^i^—Mo1—O12	108.36 (19)	O18^ii^—Mn2—O13	102.64 (14)
O18—Mo1—O12	109.8 (2)	O14—Mn2—O13	92.00 (14)
O14^i^—Mo1—O20	108.31 (17)	O18^ii^—Mn2—O1	92.34 (14)
O18—Mo1—O20	106.71 (17)	O14—Mn2—O1	99.75 (14)
O12—Mo1—O20	111.94 (16)	O13—Mn2—O1	162.10 (13)
O6^ii^—Mo2—O19	108.91 (18)	O18^ii^—Mn2—O20	176.97 (15)
O6^ii^—Mo2—O15^iii^	108.5 (2)	O14—Mn2—O20	95.05 (14)
O19—Mo2—O15^iii^	109.26 (18)	O13—Mn2—O20	78.92 (12)
O6^ii^—Mo2—O9	110.68 (17)	O1—Mn2—O20	86.59 (12)
O19—Mo2—O9	107.76 (17)	O18^ii^—Mn2—O9	92.49 (15)
O15^iii^—Mo2—O9	111.71 (17)	O14—Mn2—O9	174.66 (15)
O17^iv^—Mo3—O10^v^	109.76 (18)	O13—Mn2—O9	90.38 (13)
O17^iv^—Mo3—O3^i^	108.24 (19)	O1—Mn2—O9	79.10 (13)
O10^v^—Mo3—O3^i^	108.81 (18)	O20—Mn2—O9	90.10 (13)
O17^iv^—Mo3—O16^i^	108.86 (17)	O4—Mn3—O17	91.18 (16)
O10^v^—Mo3—O16^i^	111.28 (17)	O4—Mn3—O19^ix^	175.38 (15)
O3^i^—Mo3—O16^i^	109.85 (17)	O17—Mn3—O19^ix^	89.07 (15)
O4—Mo4—O5^i^	108.56 (18)	O4—Mn3—O10	90.76 (15)
O4—Mo4—O2^vi^	107.37 (17)	O17—Mn3—O10	90.68 (15)
O5^i^—Mo4—O2^vi^	109.08 (17)	O19^ix^—Mn3—O10	93.85 (14)
O4—Mo4—O8^i^	108.28 (18)	O4—Mn3—O16^i^	90.86 (15)
O5^i^—Mo4—O8^i^	111.05 (18)	O17—Mn3—O16^i^	177.96 (15)
O2^vi^—Mo4—O8^i^	112.35 (16)	O19^ix^—Mn3—O16^i^	88.90 (14)
O11^vii^—Mo5—O7	110.10 (18)	O10—Mn3—O16^i^	89.19 (14)
O11^vii^—Mo5—O13^viii^	106.33 (16)	O4—Mn3—O16^x^	86.67 (14)
O7—Mo5—O13^viii^	107.66 (16)	O17—Mn3—O16^x^	98.08 (15)
O11^vii^—Mo5—O1	106.51 (16)	O19^ix^—Mn3—O16^x^	88.73 (14)
O7—Mo5—O1	108.46 (16)	O10—Mn3—O16^x^	170.92 (14)
O13^viii^—Mo5—O1	117.65 (15)	O16^i^—Mn3—O16^x^	82.15 (14)
O12^iii^—Mn1—O11	93.79 (15)	O3—Mn4—O5^ii^	92.68 (16)
O12^iii^—Mn1—O20	160.75 (15)	O3—Mn4—O1	173.03 (15)
O11—Mn1—O20	105.45 (14)	O5^ii^—Mn4—O1	90.22 (14)
O12^iii^—Mn1—O7^i^	93.35 (15)	O3—Mn4—O15	96.33 (16)
O11—Mn1—O7^i^	80.40 (15)	O5^ii^—Mn4—O15	90.79 (16)
O20—Mn1—O7^i^	89.60 (13)	O1—Mn4—O15	89.96 (14)
O12^iii^—Mn1—O2	87.80 (15)	O3—Mn4—O8	86.84 (16)
O11—Mn1—O2	95.43 (14)	O5^ii^—Mn4—O8	177.36 (15)
O20—Mn1—O2	90.66 (12)	O1—Mn4—O8	89.97 (14)
O7^i^—Mn1—O2	175.73 (14)	O15—Mn4—O8	91.84 (16)
O12^iii^—Mn1—O13	83.87 (15)	O3—Mn4—O9	92.11 (15)
O11—Mn1—O13	176.15 (14)	O5^ii^—Mn4—O9	85.77 (14)
O20—Mn1—O13	76.89 (12)	O1—Mn4—O9	81.78 (13)
O7^i^—Mn1—O13	96.66 (13)	O15—Mn4—O9	171.03 (15)
O2—Mn1—O13	87.56 (13)	O8—Mn4—O9	91.65 (14)

Symmetry codes: (i) −*x*+1, −*y*+1, −*z*+1; (ii) *x*+1, *y*, *z*; (iii) *x*, *y*+1, *z*; (iv) *x*−1, *y*, *z*; (v) −*x*+1, −*y*+1, −*z*+2; (vi) −*x*+1, −*y*+2, −*z*+1; (vii) *x*+1, *y*−1, *z*; (viii) *x*, *y*−1, *z*; (ix) *x*, *y*, *z*+1; (x) *x*, *y*+1, *z*+1.
